# STAT3 signaling drives *EZH2* transcriptional activation and mediates poor prognosis in gastric cancer

**DOI:** 10.1186/s12943-016-0561-z

**Published:** 2016-12-09

**Authors:** Yuan-Ming Pan, Cheng-Gang Wang, Min Zhu, Rui Xing, Jian-Tao Cui, Wen-Mei Li, De-Dong Yu, Shu-Bin Wang, Wei Zhu, Ying-Jiang Ye, Yun Wu, Shan Wang, You-Yong Lu

**Affiliations:** 1 Key Laboratory of Carcinogenesis and Translational Research (Ministry of Education), Laboratory of Molecular Oncology, Peking University Cancer Hospital & Institute , 52 Fucheng Road, Haidian District, Beijing, 100142 China; 2 Department of Gastroenterology Surgery, Surgical Oncology Laboratory, People’s Hospital, Peking University, Beijing, 100044 China; 3 Department of Cardiology, Anzhen Hospital, Capital Medical University, Beijing, 100029 China; 4 Department of Oncology/Institute for Cancer Research, Baotou Central Hospital, Inner Mongolia, 014040 China; 5 Department of Gastroenterological Surgery, Surgical Oncology Laboratory, People’s Hospital, Beijing University, No. 11, South Xizhimen Street, Beijing, 100044 People’s Republic of China; 6 Department of Oncology/Institute for Cancer Research, Baotou Central Hospital, Baotou, 014040 People’s Republic of China

**Keywords:** EZH2, STAT3, p-STAT3, 3-deazaneplanocin A, Gastric cancer, Prognosis

## Abstract

**Background:**

STAT3 signaling plays the pivotal role in tumorigenesis through EZH2 epigenetic modification, which enhanced STAT3 activity by increased tyrosine phosphorylation of STAT3. Here, another possible feedback mechanism and clinical significance of EZH2 and STAT3 were investigated in gastric cancer (GC).

**Methods:**

STAT3, p-STAT3 (Tyr 705) and EZH2 expression were examined in 63 GC specimens with matched normal tissues by IHC staining. EZH2 and STAT3 were also identified in five GC cell lines using RT-PCR and western blot analyses. p-STAT3 protein was detected by western blotting. In order to investigate whether EZH2 expression was directly regulated by STAT3, EZH2 expression was further detected using siRNA for STAT3 or IL-6 stimulation, with dual luciferase reporter analyses, electrophoretic mobility shift assay (EMSA) and chromatin immunoprecipitation (ChIP) assays. The clinical significance of STAT3, p-STAT3 and EZH2 expression was evaluated by multi-factor COX regression and Kaplan-Meier analyses.

**Results:**

Hyper-activation of STAT3, p-STAT3 and EZH2 expression were observed in GC cells and tissues. STAT3 signaling was correlated with EZH2 expression in GC (*R* = 0.373, *P* = 0.003), which was consistent with our data showing that STAT3 as the transcriptional factor enhanced *EZH2* transcriptional activity by binding the relative promoter region (-214 ~ -206). STAT3 was an independent signature for poor survival (*P* = 0.002). Patients with STAT3^+^/EZH2^+^ or p-STAT3^+^/EZH2^+^ had a worse outcome than others (*P* < 0.001); Besides, high levels of STAT3 and EZH2 was associated with advanced TNM staging (*P* = 0.017). Moreover, treatment with a combination of siSTAT3 and EZH2-specific inhibitor, 3-deazaneplanocin A (DZNEP), increased the apoptotic ratio of cells. It is benefit for targeting STAT3-EZH2 interplay in GC treatment.

**Conclusions:**

Our results indicate that STAT3 status mediated EZH2 upregulation, associated with advanced TNM stage and poor prognosis, suggesting that combination with knockdown of STAT3 and EZH2 inhibitor might be a novel therapy in GC treatment. Collectively, STAT3, p-STAT3 and EZH2 expression were provided for the precision medicine in GC patients.

**Electronic supplementary material:**

The online version of this article (doi:10.1186/s12943-016-0561-z) contains supplementary material, which is available to authorized users.

## Background

Although the prevalence of gastric cancer (GC) has gradually decreased, it still accounts for a large portion of cancer-related deaths in China [1]. One of the most informative prognostic factors is the tumor stage, which involves both the depth of invasion and extent of metastasis. The size and histologic type of a tumor may also be useful factors in prognostication [2]. Despite the complexity of gastric tumorigenesis, several molecular studies have identified novel prognostic biomarkers. Consequently, many efforts have been made to identify and validate novel biomarkers that are not only useful for predicting prognosis and patient survival, but also for predicting the tumor response to specific anticancer drugs [3–5]. Signal transducer and activator of transcription 3 (STAT3) or enhancer of zeste homologue 2 (EZH2) is the potential molecular biomarker for tumor progression and mainly serve as the poor predictor of outcome [6–9].

Many recent studies have suggested that inflammation plays an important role in the development of GC. Aberrant IL-6/STAT3 signaling in cancer cells have emerged as a major mechanism for cancer initiation and development [10, 11]. IL-6 induces STAT3 activation, leading to cell proliferation and malignancy [9, 12, 13]. Upon activation, it is mostly involved in carcinogenesis [13, 14]. Judd et al. reported that mice with STAT3 hyperactivation developed GC in association with chronic gastritis [15]. However, it still remains unclear how constitutive activated STAT3 in GC development.

IL-6/STAT3 signaling plays an important role in regulating epigenetic aberrance during tumorigenesis, especially in the expression of certain key epigenetic enzymes, such as EZH2 [16]. EZH2, also called histone lysine methyltransferase (HKMT), was cloned as a gene belonging to the polycomb group of genes, which epigenetically silences the expression of some tumor suppressor genes (TSGs) [17]. It has been shown to be abundantly expressed in various malignancies with poor prognosis, including gastric, prostate, breast, and bladder cancers, and hematologic malignancies [6, 18–22]. Knockdown of EZH2 by siRNA has been demonstrated to inhibit breast cancer cell proliferation, whereas pharmacological inhibition of EZH2 results in the apoptosis of breast cancer cells, but not normal cells [23]. Recently, EZH2 binds to and methylates STAT3, leading to enhanced STAT3 activity by increasing tyrosine phosphorylation of STAT3 [24, 25]. The specific EZH2 inhibitor reverses the silencing of polycomb target genes and diminishes STAT3 activity. EZH2 has been shown to directly interact with and regulate the activity of the following DNA methyltransferases (DNMTs): DNMT1, DNMT3a, and DNMT3b [26, 27]. DNMTs transfer a methyl group from *S*-adenosylmethionine to the 5′-position of cytosine in CpG dinucleotides present in gene promoters, thereby maintaining a consistent pattern of epigenetic gene silencing of TSGs in cancer cells [28].

Although the genes methylated in cancer cells are packaged along with nucleosomes containing 3Me H3H27-marked genes, which are silenced in cancer, they have been shown to be independent of promoter DNA methylation, thus highlighting that 3Me H3K27 could potentially be an independent mechanism for silencing TSGs [29]. DNA methylation and transcriptional silencing of cancer genes have been shown to persist, despite the depletion of EZH2 [30], suggesting that simultaneously inhibiting EZH2 would be more effective in reversing 3Me H3K27 and DNA methylation [31]. Today, 3-deazaneplanocin A (DZNep) was reported to decrease the expression levels of the polycomb repressive complex 2 (PRC2) in cancer cells, where loss of the 3Me H3K27 marked derepression of epigenetically silenced targets [32–36]. Moreover, DZNep is a novel inhibitor of histone methyltransferase EZH2 [32, 35, 37, 38].

Previous studies have shown that increased EZH2 is involved in the pathology of gastrointestinal inflammation and associated cancers [39]. Moreover, EZH2 serves as an anti-apoptotic factor in GC development during IL-6/STAT3 activation. Taken together, it is tempting to speculate that EZH2 may be a target of STAT3 and mediate the functions of IL-6/STAT3 signaling. Until now, there is no report the potential interaction between STAT3 signaling and EZH2 during GC development. Hence, we will explore the relationship between STAT3 and EZH2 as well as other clinicopathological features in GC.

## Results

### Co-expression of STAT3 and EZH2 in GC cell lines and primary GCs

**Fig. 1:**
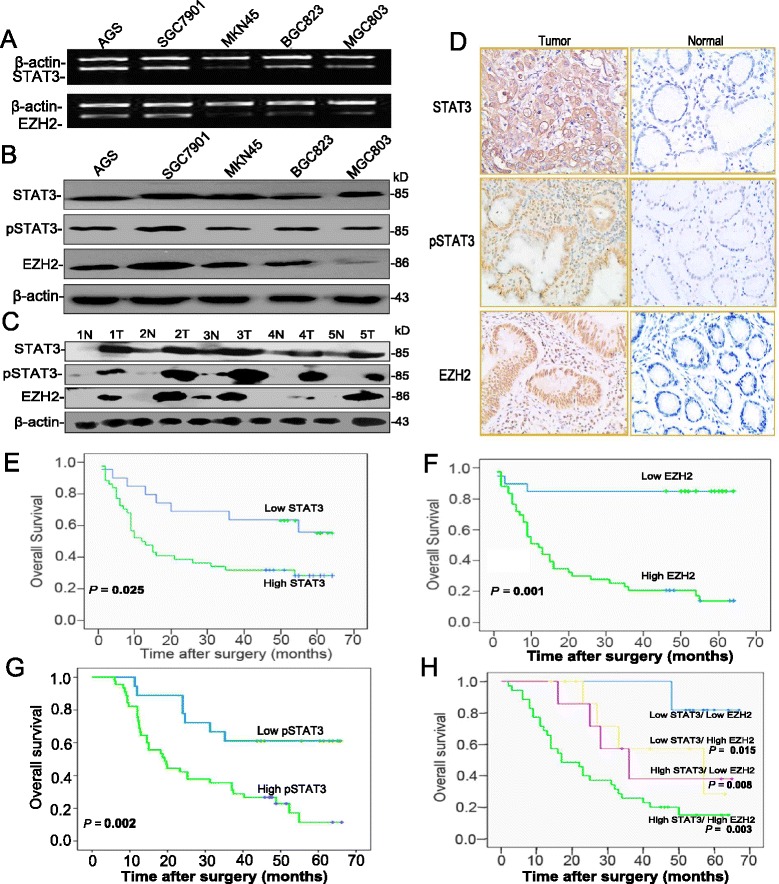
Hyperactivation of STAT3, p-STAT3 and EZH2 was associated with poor survival in the GC cohort. **a** Differential expression of STAT3 and EZH2 mRNA was detected in GC cells using RT-PCR. **b** Differential expression of STAT3, p-STAT3 and EZH2 in GC cells using Western Blotting. **c** Co-expression of STAT3 status and EZH2 protein was detected in GC and matched normal tissues using Western Blotting; **d** High or low levels of STAT3 status and EZH2 expression in GC and adjacent normal tissues using IHC staining; p-STAT3 and EZH2 showed a focal or diffuse pattern in the nuclei (200 × magnification). Kaplan-Meier analyses show the effect of STAT3 (**e**), EZH2 (**f**), p-STAT3 expression (**g**), or the combination between STAT3 and EZH2 expression (**h**) in overall survival

IL-6/STAT3 signaling plays a critical role in carcinogenesis by regulating various genes. EZH2, a protein that epigenetically silences tumor suppressor genes, was induced by IL-6 stimulation. To determine the inner relationship between STAT3 and EZH2, we first analyzed mRNA and protein expression levels of STAT3 and EZH2 in five GC cell lines using RT-PCR and real-time PCR (Fig. 1a, Additional file 1: Figure S5 and S6) and western blot analyses (Fig. 1b), respectively. All five GC cell lines expressed high STAT3 and EZH2 mRNA and protein levels. Both mRNA and protein levels were higher in SGC7901 cell lines than those in AGS, BGC823, MGC803, and MKN45 cell lines. Western blot analyses revealed two immunoreactive signals, an 85-kDa for STAT3 and an 86-kDa for EZH2.

The levels of STAT3, p-STAT3 and EZH2 expression in GC tissues and their corresponding non-cancerous gastric mucosa were analyzed by Western blot. The levels of STAT3 status and EZH2 expression in GC were higher than those in normal tissues (Fig. 1c). IHC staining showed that higher level of STAT3 expression in GC tissues (43/63, 68.2%) than that in the corresponding normal tissues (24/63, 38.1%, *P* = 0.003; Table 1 and Fig. 1d). Intense nuclear staining was observed for EZH2 in GC (Fig. 1d). As shown in Table 1, the protein expression of EZH2 in the nuclei of GC tissues was significantly higher (47/63; 74.6%) than those in normal tissues (21/63, 33.3%, *P* = 0.001). A close correlation between STAT3 and EZH2 was observed in the cohort by χ^2^-test (Spearman rank correlation coefficient = 0.373, *P* = 0.003; Fig. 1d; Additional file 1: Table S2).

**Table 1:** Proportion of cases according to EZH2 and STAT3 protein expression

Group	Cases n	EZH2 expression		STAT3 expression	
		Low/- (%)	High/+ (%)	Low/- (%)	High/+ (%)
Tumor	63	16 (25.3)	47 (74.6)	20 (31.7)	43 (68.2)
Normal	63	42 (66.7)	21 (33.3)	39 (61.9)	24 (38.1)

EZH2, *P* = 0.001; STAT3, *P* = 0.003

### Co-expression of STAT3 and EZH2 correlated with poor survival in GC patients

A significantly elevated expression of STAT3 and EZH2 was noted in 43 (68.2%) and 47 (74.6%) cases, respectively. Furthermore, 82.5% (52/63) of the patients were either EZH2^+^ and/or STAT3^+^, of which 35 (55.6%) showed a combined positivity for STAT3 and EZH2 (Additional file 1: Table S2). Notably, more than half the patients belonged to the high-expression group.

Univariate analysis demonstrated that high expression of EZH2 was significantly associated with poor survival (*P* = 0.001, log-rank; Fig. 1f). In multivariate Cox-regression analyses, STAT3 was a significant independent predictor of survival (HR: 7.535; 95% CIs: 2.104 ~ 26.993; *P* = 0.002; Additional file 1: Table S4). In contrast, EZH2 was not an independent factor in GC prognosis (HR: 0.863; 95% CIs: 0.337 ~ 2.204; *P* = 0.757; Additional file 1: Table S4).

Further supporting these results, the overall survival (OS) rate was significantly correlated with STAT3 protein expression. In this cohort, the 5-year OS rate was 31.6%. As expected, the OS was significantly higher in the STAT3^−^ group than in the STAT3^+^ group (*P* = 0.025, log-rank test; Fig. 1e). The OS of pSTAT3^−^ group was also better than pSTAT3^+^ group (*P* = 0.002, log-rank test; Fig. 1g). Furthermore, it was observed that OS was better in the STAT3^−^/EZH2^−^ group than in other groups. In particular, the 5-year OS rate was significantly lower in STAT3^+^/EZH2^+^ patients (22.3%) than in STAT3^−^/EZH2^−^ patients (52.4%, *P* < 0.001). Additionally, patients with EZH2^+^/STAT3^+^ expression had poorer prognosis than those with either EZH2^−^ and/or STAT3^−^ expression (*P* = 0.007, log-rank; Fig. 1h). Similar results were shown in the combination between p-STAT3 and EZH2 expression (Additional file 1: Figure S3).

Therefore, these results indicate that STAT3 and EZH2 are important genetic markers in predicting a poor prognosis for GC patients undergoing resection. Thus, our findings highlight the value of EZH2 as a predictor of survival that could be more significant when considered in conjunction with STAT3 in GC.

### Co-expression of STAT3 and EZH2 correlated with TNM stage in GC

In addition, most of GC samples exhibited coexpression of STAT3 and EZH2 in the cohort (Fig. 1c). Furthermore, the association of STAT3 and EZH2 immunohistochemical expression levels with clinicopathological features was evaluated in 63 GC samples (Table 2). STAT3 expression in GC tissues was found to be significantly associated with patients’ age (*P* = 0.024), TNM stage (*P* = 0.0001), and lymph node metastasis (*P* = 0.016), and EZH2 expression was positively correlated with patients’ gender (*P* = 0.043) and TNM stage (*P* = 0.002). The present study showed that hyperactivation of STAT3 and EZH2 in GC tissues was significantly associated with advanced TNM stage. Notably, 40% of GC tissues, corresponding to TNM stages I and II, and 65.7% of GC tissues, corresponding to TNM stages III & IV, showed a significantly high expression of both STAT3 and EZH2 (Additional file 1: Table S3, *P* = 0.017).

**Table 2:** Correlation between EZH2, STAT3 and clinicopathologic features in GC

Characteristics		Cases n	EZH2 expression		*P*-value	STAT3 expression		*P*-value
			Low/-	High/+		Low/-	High/+	
Age (y)	≤60	22	6	16	0.804	11	11	0.024
	> 60	41	10	31		9	32	
Gender	Male	47	15	32	0.043	17	30	0.200
	Female	16	1	15		3	13	
Differentiation	Well	11	5	6	0.164	3	8	0.658
	Moderate	13	4	9		3	10	
	Poor	39	7	32		14	25	
Borrmann’s pathologic classification	Ulceration	47	10	37	0.342	15	32	0.678
	Prominence	7	2	5		3	4	
	Diffuse	9	4	5		2	7	
Tumor Location	Antrum	45	11	34	0.785	14	31	0.865
	Body & Cardia	18	5	13		6	12	
TNM stage	I	12	8	4	0.002	10	2	0.0001
	II	13	4	9		3	10	
	III	27	3	24		7	20	
	IV	11	1	10		0	11	
Lymph node metastasis	No	30	12	18	0.051	14	16	0.016
	Yes	33	4	29		6	27	
Distant metastasis	No	53	13	40	0.738	18	35	0.388
	Yes	10	3	7		2	8	

### STAT3 signaling enhances *EZH2* promoter activity in GC cells

**Fig. 2:**
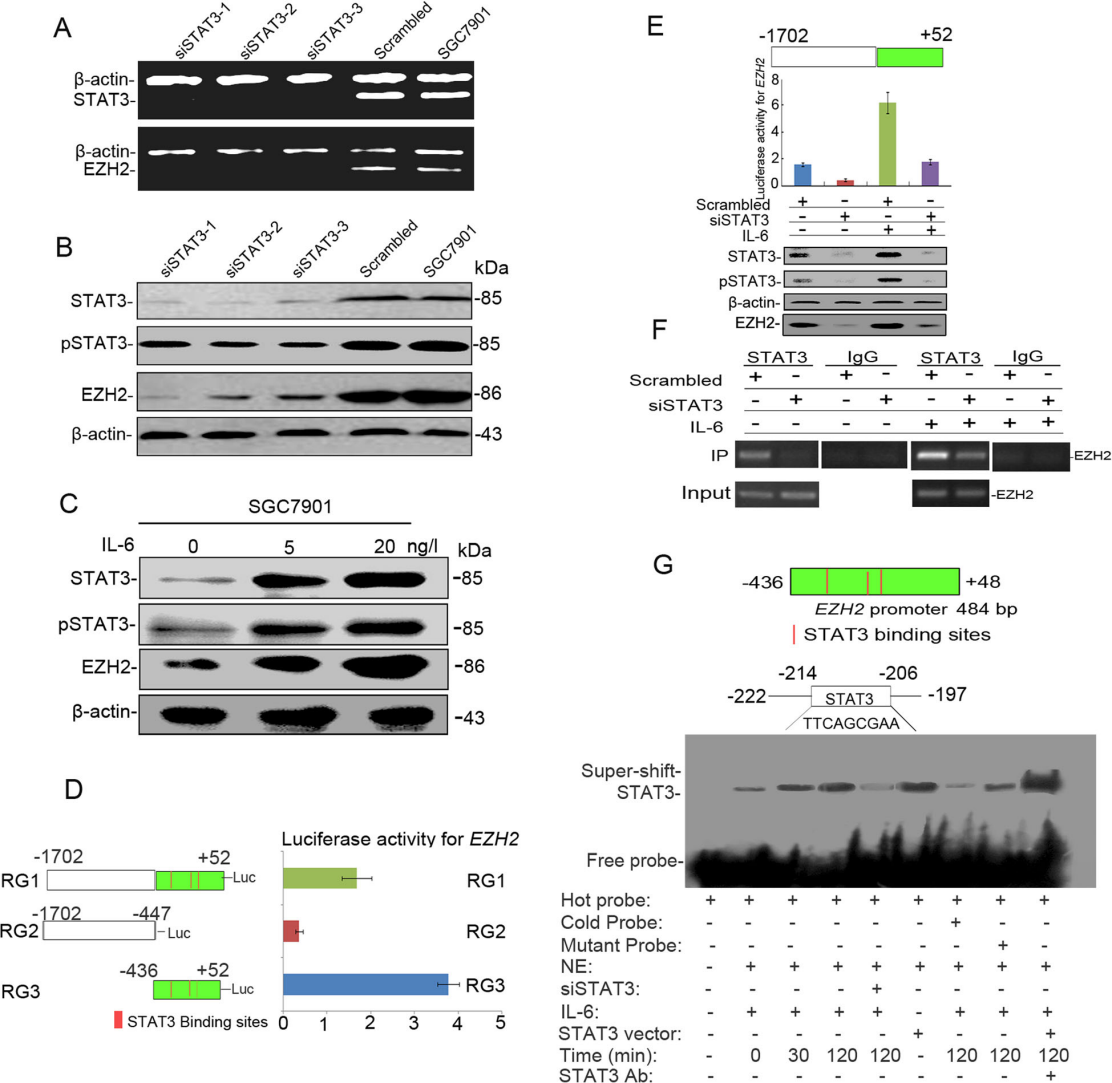
EZH2 is a potential downstream target of STAT3 signaling. **a** EZH2 mRNA expression was decreased in SGC7901 cells transfected with siSTAT3. **b** The protein level of EZH2 expression was downregulated in SGC7901 cells using knockdown of STAT3 with siRNA. **c** The expression of STAT3 status and EZH2 was induced by IL-6 in SGC7901 cells. **d** Luciferase activity was measured in extracts from SGC7901 cells transfected with different luciferase reporter constructs, containing the full-length promoter (Region 1) or the region only containing STAT3-binding sites (Region 3) or not (Region 2); luciferase activity normalized for *Renilla* luciferase activity and expressed relative to the activity of the untreated group; the higher activity of EZH2 was detected in Region 1 and Region 3, which contained STAT3-binding sites (Fig. 2d, *P* < 0.001). **e** Dual luciferase reporter analysis of *EZH2* promoter. The construct with full-length of *EZH2* promoter (-1702/+52) was inactivated by siSTAT3 treatment with or without IL-6 stimulation. **f** The specific region (−436/+48) of EZH2 promoter was detected by ChIP-PCR. STAT3 mediated fold-enrichment of STAT3-binding regions of *EZH2* promoter. Further, the binding activity was increased by IL-6 stimulation compared with the untreated group (*P* = 0.0059). When knockdown of STAT3 using siRNA, the binding activity was decreased with or without IL-6 (*P* = 0.0043). **g** The nuclear extracts from SGC7901 cells were incubated with biotin-labeled wild-type before the mutant or cold probes were added for 20 min. The specific region of the STAT3 stand motif (−222/−197) demonstrated effective binding ability. All the results for *EZH2* activity were analyzed by EMSA. The results in (**d**) and (**e**) are represented as mean ± SD values

Given the co-expression of STAT3 and EZH2 in GC, we investigated whether STAT3 could regulate the expression of EZH2; thus, we analyzed EZH2 expression at both mRNA and protein levels in SGC7901 cells transfected with three pairs of siSTAT3 primers and scrambled negative control siRNA. Interestingly, STAT3 siRNAs decreased the level of STAT3 and EZH2 expression (Fig. 2a and b, Additional file 1: Figure S4). And the high levels of STAT3 and EZH2 were induced by IL-6 stimulation (Fig. 2c), subsequently, siRNA of STAT3 after IL-6 addition, the luciferase reporter was reduced at the original level of background (Fig. 2e). Our results indicated that EZH2 was a potential target gene of STAT3 signaling.

We performed transient expression studies in order to explore the effect of STAT3 signaling on *EZH2* promoter activity. The level of *EZH2* promoter activity in siSTAT3-treated SGC7901 cells was found to be significantly lower than that in the untreated control. The relative activity of EZH2 was decreased by siSTAT3 (*P* = 0.0248), but it was increased to 6.18-fold by IL-6 stimulation (*P* = 0.0035; Fig. 2e). Combined siRNA for STAT3 with IL-6 addition, decreased activity of *EZH2* promoter luciferase reporter was detected apparently compared with IL-6 stimulation alone in SGC7901 cells. Our study highlights the potential interplay that STAT3 signaling promotes EZH2 expression in GC cells.

We had also performed a detailed analysis of the *EZH2* promoter in the NCBI database, and identified that it contained three conserved STAT3-binding sites at the main promoter region of *EZH2* gene (Additional file 1: Figure S1). STAT3 binds to two known sequences, HIS and GAS, to exert its anti-apoptotic and oncogenic effects. These sites contain the canonical STAT3-binding motifs TTC(N)_2-4_GAA or TT(N)_4-6_AA [40]. Hence, we determined that the STAT3-responsive elements are present in the *EZH2* promoter at position −346 to +52, which, in turn, corresponds to the consensus STAT3-binding site TTN_(4-6)_AA. Corroborating these findings, the results of our study demonstrated a significant decrease in luciferase activity for the shorter length *EZH2* promoter gene (−436 to +52), as compared to that of the full length *EZH2* promoter (−1702 to +52; Fig. 2d, *P* = 0.024; Additional file 1: Figure S1), indicating that the promoter region between −436 and +52 is critical for *EZH2* promoter activation in response to STAT3. This fragment contains the 3 STAT3-binding motifs described above.

Subsequently, we performed ChIP-PCR analysis using SGC7901 cells to determine the precise consensus sequences for *EZH2* promoter activation and to further investigate the role of the promoter fragment −436 to +52 containing three motifs of STAT3. Furthermore, in order to confirm that STAT3 bound to the *EZH2* specific promoter_(-436-- + 52), we used a ChIP-PCR procedure, comprising 33 PCR cycles optimized to achieve amplification of DNA that had been precipitated with STAT3. In the absence or presence of siSTAT3 after IL-6 stimulation or not, the enrichment of STAT3-binding to *EZH2* fragments was decreased after knocking down of STAT3 by PCR and quantitative real-time PCR analyses (Fig. 2f, Additional file 1: Figure S7), coinciding with the downregulation of EZH2 expression at mRNA and protein levels (Fig. 2a, and b). Our results demonstrated that STAT3 was recruited to *EZH2* promoter region at the main three STAT3-binding motifs, which indicates that *EZH2* transcription is required for STAT3-binding enrichment.

Three STAT3 *cis*-element sequences, STAT3-1, STAT3-2 and STAT3-3, were located at different regions of the *EZH2* main promoter. To further confirm the effect of STAT3 fragments on the transcriptional activity of *EZH2* promoter, four vectors were constructed, including vector p373 (Fragment 1) containing the three Stat3-binding fragments, vector p222 (Fragment 2) lacking the Stat3-1 fragment, vector p163 (Fragment 3) lacking both Stat3-1 and STAT3-2 fragments, and vector p131 (Fragment 4), which has no Stat3-fragment (Additional file 1: Figure S2). Transfection of p373 (Fragment 1) and p222 (Fragment 2) led to an increase in luciferase activity compared with the transfection of p163 (Fragment 3) and p131 (Fragment 4), especially, the luciferase activities of Fragment 1 and Fragment 2 were higher than Fragment 3 and 4 (Fragment 1: 4.67 ± 0.56; Fragment 2: 4.86 ± 0.68; Fragment 3: 1.12 ± 0.27; Fragment 4: 0.74 ± 0.16; *P* = 0.0063; Additional file 1: Figure S2). And there was no obvious different between Fragment 1 and 2 for the *EZH2* luciferase activity (*P* = 0.094; Additional file 1: Figure S2). It means that the second Stat3 motif was important for regulation of EZH2. These data narrowed the enhancer activity to the -222 bp to -163 bp region, and suggested that this Stat3-binding region contained a *cis*-acting element that interacted with STAT3 to induce transcription.

To investigate which Stat3 motif region binding to *EZH2* promoter, an EMSA was performed using synthetic 26-bp oligo-nucleotides containing the Stat3-binding fragment (Additional file 1: Table S1). We examined the binding activity of the nuclear extract to candidate nucleotide sequences in order to identify the STAT3-responsive element. Several possible permutations of the STAT3-binding site were also systematically synthesized and tested for their ability to bind activated STAT3. The results showed that STAT3 enhanced the binding activity of nuclear extracts to the probe (containing Stat3 motif). The synthesized mutant sequences were found to show little or no binding to activated STAT3. Further experiments with the wild-type probe, without biotin modification, which “cold-competed” in the EMSA assay, demonstrated a significant decrease in the STAT3 target-binding capacity *in vivo* (Fig. 2g), it indicated that stat3-binding site located in the -214 bp to -206 bp region, played the important role in the transcriptional activity of *EZH2* gene.

### Anti-apoptotic activity of STAT3-mediated EZH2 in GC cells

**Fig. 3:**
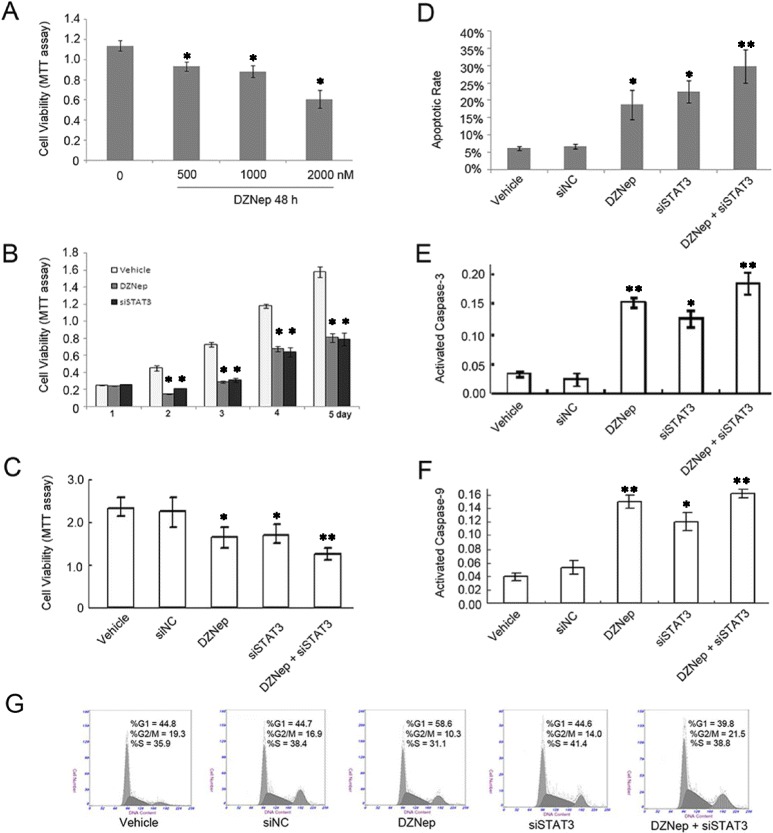
EZH2 mediated anti-apoptotic effects in STAT3 signaling pathway. siSTAT3 and EZH2 inhibitor, DZNep, suppressed cell proliferation through apoptosis and cell cycle arrest. **a** DZNep, as the specific EZH2 inhibitor, induced cell apoptosis with a dose-dependent manner (*P* = 0.0064). **b** siSTAT3 and/or DZNep (500 nM), decreased cell viability, as determined by MTT assay (*P* = 0.0052). **c** Effects of siSTAT3 transfection and/or DZNep exposure on cell viability for 48 h by MTT assay. Decreased cell viability was clearly detected in the group treated with DZNep and/or siSTAT3 compared with the relative negative control (DZNep vs. Vehicle (0.1% alcohol, solution for DZNep), *P* = 0.0183; siSTAT3 vs. siNC, *P* = 0.0026, DZNep + siSTAT3 vs. Vehicle, *P* < 0.001; DZNep + siSTAT3 vs. siNC, *P* < 0.001). **d** The apoptotic rate was increased upon DZNep treatment and/or siSTAT3 transfection by FITC-Annexin-V/PI staining (DZNep vs. Vehicle, *P* = 0.0023, siSTAT3 vs. Vehicle, *P* =0.0035; DZNep + siSTAT3 vs. Vehicle, *P* = 0.001, respectively). **e** Caspase-3 activity in cells treated with SGC7901, siNC, DZNep, siSTAT3, or a combination of DZNep and siSTAT3. The activity of caspase-3 was higher in the group treated with siSTAT3 and/or DZNep treatment than in cells treated with vehicle (DZNep vs. Vehicle, *P* = 0.0056; DZNep + siSTAT3 vs. Vehicle, *P* = 0.0029) and siNC (siSTAT3 vs. siNC, *P* = 0.0215; DZNep + siSTAT3 vs. siNC, *P* = 0.0038). **f** Caspase-9 activity in cells treated with SGC7901, siNC, DZNep, siSTAT3, or a combination of DZNep and siSTAT3. The activity of caspase-9 was higher in the group treated with siSTAT3 and/or DZNep treatment than in cells treated with vehicle (DZNep vs. Vehicle, *P* = 0.0033; DZNep + siSTAT3 vs. Vehicle, *P* = 0.0012) and siNC (siSTAT3 vs. siNC, *P* = 0.0137; DZNep + siSTAT3 vs. siNC, *P* = 0.0214). **g** Cell cycle remained unchanged treated by DZNep alone in cells. All results in (**a**), (**b**) and (**c**) are presented as the mean ± SD values of three assays

Aberrant expression of STAT3 is known to contribute to malignancy [12]. In the present study, inhibition of STAT3 signaling by siRNA significantly increased caspase-3/9 positivity in SGC7901 cells (Fig. 3e and f), suggesting that STAT3 contributes to the anti-apoptotic effect in GC cells (Fig. 3d). We further evaluated the intracellular changes in GC cells treated with the EZH2-specific inhibitor, DZNep. We observed that DZNep reduced the expression of EZH2, which resulted in increased caspase-3/9 activity (Fig. 3e and f), leading to apoptosis of SGC7901 cells (Fig. 3a, b and c). The single agent DZNep treatment did induce G1/G0 phase arrest in our study (Fig. 3g, *P* = 0.0038). Thus, our findings demonstrate that EZH2 is the downstream target gene of STAT3 signaling and plays an important role in the anti-apoptotic effect of the latter in GC cells.

In addition, our clinical data showed that hyperactivation of STAT3 and EZH2 occurred in GCs. Further supporting this clinical observation, knockdown experiments involving the treatment of GC cells with STAT3 siRNA in the presence of DZNep demonstrated an increased apoptotic rate, as well as enhanced caspase-3/9 activity (Figs. 3e and f, *P* = 0.003, *P* = 0.027, respectively), which in turn resulted in the down-regulation of EZH2 at both mRNA and protein levels. We next examined cell cycle variation and apoptosis in cells treated with siSTAT3 or DZNep alone or in combination. As shown in Fig. 3g, cells treated with DZNep alone can induce apoptosis and G1/G0 phase arrest; the combination of STAT3-siRNA and DZNep induced more apoptosis than others (*P* < 0.05; Fig. 3c). It will be important that these trials can measure high-resolution DNA methylation and histone modifications in order to correlate clinical responses with candidate epigenetic changes.

## Discussion

Several studies have suggested that STAT3 and EZH2 are closely associated with cell proliferation, invasion, and metastasis [37–40]; our findings demonstrate that co-expression of STAT3 and EZH2 in GC cells is associated with poor prognosis. In particular, the activation of EZH2 and STAT3 is significantly correlated to TNM stage and patient survival, suggesting that a combination of STAT3 and EZH2 expression could determine clinical TNM stage and predict disease outcome.

STAT3 is positively correlated with EZH2 expression in GC cells and tissues. Knockdown of STAT3 resulted in down-regulation of EZH2 at the mRNA and protein levels. We next determined whether STAT3 could be a transcriptional regulator of the *EZH2* gene. We transfected SGC7901 GC cells with a reporter vector encoding luciferase under control of the *EZH2* promoter. Knockdown of STAT3 by siRNA decreased *EZH2* promoter activity, which was abrogated by mutation of the STAT3 DNA-binding site in the *EZH2* promoter. We next carried out ChIP assay to assess whether STAT3 could directly bind to the *EZH2* promoter. STAT3 protein binding at the *EZH2* promoter was significantly increased in SGC7901 cells, and was increased 6.18-fold with IL-6 stimulation, which is consistent with the results of studies showing that EZH2 protein expression is induced by IL-6 in multiple myeloma cell lines [39]. As expected, transfecting cells with a siSTAT3 significantly decreased luciferase reporter activity. Upon further study, we found three STAT3 *cis*-element-binding sites in the EZH2 promoter. Deletion analysis showed that the second STAT3-binding site was active in the luciferase reporter assay following IL-6 stimulation. Mutation of this STAT3-binding motif decreased its ability to bind STAT3. This study demonstrates that STAT3 directly regulates EZH2 expression by binding to *EZH2* promoter, which is consistent with the results of the study in CRC [41].

Furthermore, down-regulation of STAT3 promoted apoptosis through the suppression of EZH2, and the activated caspase-3/9 were detected. Our results further strengthened the results of previous studies that demonstrated an anti-apoptotic effect of EZH2 and STAT3 over-expression via the Akt/Bad/Bcl-xL apoptotic pathway [8, 42]. The present study was first to demonstrate an underlying mechanism involving the regulation of EZH2 by STAT3 and to propose the existence of a positive functional loop between STAT3 and EZH2. The functional relationship between EZH2 and STAT3 could be the mechanism by which STAT3 regulates cell proliferation. Indeed, DZNep, an EZH2-specific inhibitor, increased apoptosis of GC cells, when combined treatment with DZNep and siSTAT3 further increased the apoptotic rates in GC cells.

**Fig. 4:**
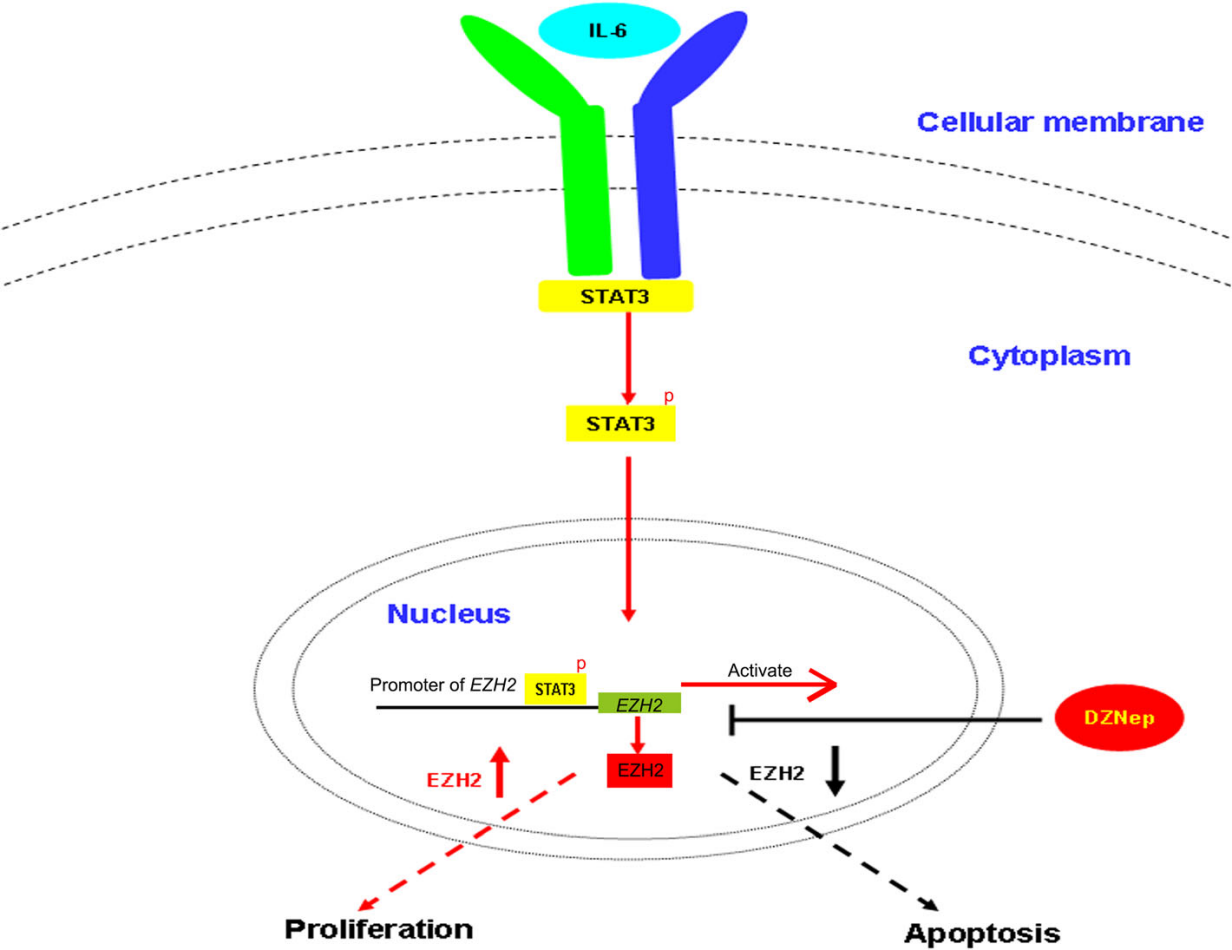
The possible mechanism of STAT3 in regulating EZH2 expression

In conclusion, our study identified EZH2 protein as an important molecule downstream of STAT3, which mediates an anti-apoptotic effect in concert with STAT3 (Fig. 4). Our findings suggest that EZH2 possesses anti-apoptotic activity in gastric tumorigenesis following STAT3 activation. Further studies are required to elucidate the detailed functional roles of these molecules in order to exploit them as candidates for new therapeutic targets.

## Conclusion

We found that both mRNA and protein expression levels of EZH2 were decreased by knocking down of STAT3 with siRNA in GC cells. Moreover, STAT3, the transcriptional factor, induced *EZH2* activation by binding to the specific Stat3 motif of *EZH2* promoter (-214 ~ -206). High levels of EZH2, STAT3 and p-STAT3 expression were significantly associated with poor prognosis in GC patients. Furthermore, combined EZH2 and STAT3 or p-STAT3 was apparently associated with worse clinical outcome, suggesting that the panel of EZH2 and STAT3 could be served as the potential of molecular prognostic signature. Our study also found that treatment of GC cells with STAT3-siRNA and/or the presence of EZH2 specific inhibitor, DZNep, enhanced the downregulation of EZH2 expression with increasing apoptosis. Thus, combination between siSTAT3 and EZH2 inhibitors could be contributed for the potential epigenetic therapy against GC patients.

## Methods

### Reagents and cell cultures

Human IL-6 was purchased from Roche (Basel, Switzerland). Antibodies, including anti-STAT3, anti-p-STAT3 (Tyr 705), anti-EZH2, and anti-β-actin were obtained from Cell Signaling Technology (Beverly, MA), Abgent (San Diego, CA), and Sigma (St Louis, MO), respectively. DZNep (Cayman Chemical, Michigan) was used as EZH2-specific inhibitor. Five human GC cell lines were employed. The cell lines BGC823, MGC803, SGC7901 were purchased from the Cell Bank of Shanghai (Shanghai, China). The MKN45 and AGS cell lines were purchased from American Type Culture Collection (ATCC; South San Francisco, CA). Cells were cultured in Dulbecco’s modified Eagle’s medium (DMEM; Life Technologies, Grand Island, NY, USA) with 10% fetal bovine serum (FBS; Life Technologies) and were maintained in a humidified 5% CO_2_ incubator at 37 °C.

### Patients and tissue specimens

The study was scrutinized and approved by the Hospital Bioethics Committee, and patient consent was obtained prior to the initiation of the study. The prospective study group comprised 63 patients who had primary gastric adenocarcinomas and underwent gastrectomy between January and December in 2008 at the Department of Gastroenterology Surgery, Surgical Oncology Laboratory, Beijing Cancer Hospital and People’s Hospital. The inclusion criteria for the study were as follows: (a) the patient had no concurrent diseases precluding the administration of systemic chemotherapy, and (b) the patient had not received preoperative radiotherapy. All patients were followed-up prospectively for a maximum period of 66 months. Tissue samples of GC as well as adjacent non-cancerous (normal appearance) gastric tissues were fixed in 10% neutral formalin, processed for paraffin sections, and used for histopathology and immunohistochemistry (IHC) studies. Clinicopathological information was obtained from medical charts, and histopathological examination was performed according to the 6^th^ edition of the American Joint Committee on Cancer System (AJCC) staging system [41, 43]. All available H&E-stained slides of the surgical specimens were reviewed.

### Immunohistochemical analysis

Formalin-fixed paraffin-embedded sections (4-μm-thick) from samples were collected for IHC experiments. STAT3 and EZH2 were detected using rabbit polyclonal antibodies. Briefly, sections were incubated with rabbit anti-STAT3 and anti-EZH2 (1:200) overnight at 4 °C. Normal goat serum was used as a negative control. After washing, tissue sections were treated with biotinylated anti-rabbit secondary antibody (Santa Cruz, CA) followed by further incubation with streptavidin-horseradish peroxidase complex (Dako, Carpinteria, CA), and then immersed in 3,3-diaminobenzidine, counterstained with 10% Mayer’s hematoxylin, dehydrated, and mounted. Both nuclear and cytoplasmic staining was observed for STAT3, while only nuclear staining was seen for EZH2. The intensity of immunoreactivity was assessed for EZH2 and STAT3 as follows: high expression, ≥ 50% cells showing intense immunoreactivity; low expression, < 50% cells showing intense immunoreactivity. The mean percentage of positive tumor cells was determined in at least five areas using a high-power field microscopy. Immunopositivity was independently evaluated by two senior pathologists.

### RNA extraction, reverse transcriptional PCR and real-time PCR analyses

Total RNA was extracted from cells by using Trizol reagent (Invitrogen, Life Technologies) according to the manufacturer’s instructions. The extracted RNA was pretreated with RNase-free DNase, and 5 μg RNA from each sample was used for cDNA synthesis primed with random hexamers. PCR amplification of STAT3 or EZH2 cDNA using STAT3 or EZH2-specific primers (Additional file 1: Table S1) was performed under the following conditions: initial denaturation step at 95 °C for 5 min; followed by 30 cycles consisting of denaturation at 95 °C for 30 s, primer annealing at 60 °C for 30 s, primer extension at 72 °C for 30 s; and a final extension at 72 °C for 5 min. β-actin was used as an internal control. Furthermore, these gene expression was running by ABI 7500 Fast Real-time PCR System (Applied Biosystems, Carlsbad, CA, USA) with SYBR green (TransGen Biotech Co., Ltd., Beijing, China). 1 μl cDNA and 1 μl primers were mixed to the final volume of 12 μl. The final q-PCR conditions were described briefly: a pre-denaturation at 95 °C for 20 s, followed by 40 cycles at 95 °C for 3 s and extension at 60 °C for 30 s. The endogenous control was β-actin. Each sample was performed in triplicate.

### Western blotting

Cells were lysed in buffer containing 50 mM Tris-HCl (pH7.4), 125 mM NaCl, 0.1% Triton-X, and 5 mM EDTA, and treated with 1% (v/v) protease inhibitor and 1% (v/v) phosphatase inhibitor cocktail II (Sigma). Subsequently, 50 μg total protein from each cell-lysate was separated by SDS-PAGE, followed by electrotransfer onto a PVDF membrane (Millipore, Billerica, MA, USA). Rabbit polyclonal antibodies against STAT3 (1:1000 dilution) and EZH2 (1:600 dilution) were used as primary antibodies; anti-β-actin (1:10,000 dilution) was used as a control. Horseradish peroxidase-conjugated anti-rabbit/mouse IgG antibody (Amersham Biosciences, Piscataway, NJ) was used as a secondary antibody. Immunodetection was accomplished using enhanced chemiluminescence (Amersham Biosciences).

### MTT assay

SGC7901 cells were seeded into 96-well culture plates, and 3-(4,5-dimethylthiazol-2-yl) -2,5-diphenyltetrazolium bromide (MTT) was added to each well containing cells treated with DZNep and/or siRNA mimics for STAT3 (siSTAT3) from 24 to 120 h; plates were then further incubated for 4 h at 37 °C. Subsequently, MTT was removed and dimethyl sulfoxide (DMSO, Sigma) was added to the formazan product. The absorbance was read at 490 nm/570 nm using microplate readers (Bio-Rad 680, Life Science, Hercules, CA, USA).

### Flow cytometry analysis for apoptosis

SGC7901 cells were treated with DZNep and or siSTAT3 for 48 h, followed by harvesting, counting (1 × 10^6^ cells) and re-suspending in 100 μl of phosphate-buffered saline (PBS). Afterward, 5 μl of Annexin V (1 μg/ml) (Beckman Coulter, Fullerton, CA) was added and incubated at RT for 15 min, then 10 μl of propidium iodide (PI, 1 μg/ml) was added and incubated for additional 5 min at room temperature in the dark. Finally, the cells were subjected to flow cytometry (FCM) to measure the apoptosis rate with an Epics-XL-MCL flow cytometer (Beckman Coulter, USA).

### Flow cytometry analysis for cell cycle

Cell cycle analysis was performed using flow cytometry employing a Cell Cycle Detection kit, according to the manufacturer’s instructions (KeyGEN Biotech, Nanjing, China). The SGC7901 cells, were treated with DZNep (500 μM) and/or siSTAT3 mimics for 48 h. Subsequently, the cells were sorted using a FACSCalibur (BD Biosciences, Franklin Lakes, NJ, USA), and cell-cycle profiles were analyzed using WinMDI v 2.9 software (Scripps Research Institute, La Jolla, CA, USA).

### Promoter and Luciferase reporter assays

Luciferase assays were performed using the Dual Luciferase Reporter Assay System (Promega, Madison, WI, USA). Promoter constructs for the assays were generated by cloning the region of the human *EZH2* promoter from −1702 to +52 and inserting it between the *Sac*I and *Xho*I restriction sites of the pGL3-Basic vector (Promega). After cloning and confirmation of the nucleotides of the *EZH2* promoter by sequencing, the construct was named *EZH2*-promoter-Luc (Additional file 1: Table S1).

SGC7901 cells were co-transfected with 800 ng of *EZH2*-promoter-Luc and 6 ng *Renilla* luciferase plasmid pRL-TK using Lipofectamine 2000 transfection reagent (Life Technologies), with or without IL-6 treatment (1000U/ml). Cells were also transfected with 300 ng of siSTAT3 to inhibit STAT3 signaling (Life Technologies). At 24 h after transfection, the cells were washed, lysed, and evaluated sequentially for firefly luciferase and *Renilla* luciferase activities (Promega) using a BD Monolight 3010 luminometer (BD Biosciences) or a Lumat luminometer (LB 9507, Germany). The results obtained were normalized for *Renilla* luciferase activity and expressed relative to the activity of the untreated cell group transfected with *EZH2*-promoter-Luc. Promoter activity was reported as mean ± SD values.

#### Chromatin immunoprecipitation (ChIP) and quantitative real-time PCR assay

SGC7901 parent cells or knockdown of STAT3 in cells, followed by stimulation with or without IL-6 (1000 U/ml), were fixed with 1% formaldehyde and lysed for 10 min at 37 °C, and sonicated to obtain sheared DNA fragments of approximately 200 ~ 1000 bp. The chromatin was then incubated and precipitated with antibodies against the STAT3 antibody or IgG (Santa Cruz), after which DNA-protein immunocomplexes were collected, using protein A/G-agarose beads (Pierce Biotechnology, Rockford, IL, USA), and treated with RNase A (Sigma) and proteinase K (Sigma), primers were designed for Stat3-ChIP enriched the promoter region of *EZH2* gene. Real-time PCR was performed on non-amplified Stat3, rabbit IgG, and Input of SGC7901 cells. ChIP DNA-enrichments was used SYBR Green Master Mix reagents with an ABI PRISM 7900HT sequence detection system (pre-denaturation at 95 °C for 5 min, followed 95 °C for 10 s, 60 °C for 10 s, 72 °C for 30 min, 40 cycles). The ready-to-use primers were employed for studying transcriptional regulation of *EZH2* at or around its transcriptional start site (TSS). The enrichment of Stat3 motifs binding at or around the TSS of *EZH2* could be reliably detected and quantified by ChIP real-time PCR assay or the PCR products were run on 2% agarose gel in 1× TBE buffer.

### Electrophoretic mobility shift assay (EMSA)

EMSA nuclear extracts were prepared using the Applygen protocol (Applygen Technologies Inc., Beijing, China). For the mobility shift assay for STAT3-DNA binding activity, a nucleotide sequence corresponding to the 5′-flanking region of human *EZH2*, containing three conserved STAT3-binding motifs, was used (Additional file 1: Figure S1). The EMSA probe contained the second conserved STAT3-binding motif (Additional file 1: Table S1). DNA probes for EMSA were synthesized as oligonucleotides (Sangong, Shanghai; Additional file 1: Table S1). The “hot probe” was generated by labeling the 5′-end with biotin. Furthermore, we employed a “cold probe” or “mutation probe”, which lacked 5′-biotin labeling, in the competitive EMSA to assess the involvement of STAT3 (Additional file 1: Table S1). SGC7901 cells were pre-treated with or without STAT3 siRNA, and nuclear protein was extracted as described previously.

SGC7901 cells were pre-treated with or without siSTAT3, and nuclear protein was extracted. EMSA was carried out with a Gel Shift assay System (Promega) in accordance with the manufacturer’s recommendation. Nuclear protein (10 μg) was pre-incubated in a final volume of 15 μl of buffer containing 10 mM Tris-HCl (pH 7.5), 1 mM MgCl_2_, 50 mM NaCl, 0.5 mM EDTA, 4% glycerol, 0.5 mM DTT, and 0.5 mg of poly (dI:dC) for 10 min, and the biotin-labeled probe was added to the mixture; samples were incubated for 20 min at room temperature. The protein-DNA complexes were then electrophoresed on a 7% acrylamide gel and analyzed by autoradiography.

### Apoptosis and caspase assay

Annexin V-FITC/PI analysis (BD Bioscience, Franklin Lakes, NJ, USA) was used to measure apoptosis induction in the siSTAT3 transfected groups with or without the EZH2 inhibitor, DZNep treatment. Harvested cells were washed twice in buffer and resuspended at a concentration of 5 × 10^5^ SGC7901 cells in 1 ml of buffer containing at least 40 mM Ca^2+^. Cells were then added to a tube containing 5 ml of fluorescent-Annexin V/PI. Fluorescence was quantified on a Becton Dickinson FACScan flow cytometer (BD Biosciences) for at least 10,000 events.

After cells were transfected with siSTAT3 (or scrambled siRNA), they were cultured for 12 h in 6-cm dishes containing serum-free medium with or without DZNep. The cells were washed with 1× PBS and resuspended in lysis buffer. Caspase-3/9 activity was assessed using a Colorimetric CaspACE™ assay System (Promega). The lysate was mixed with Z-DEVD-*p*NA and Z-LEHD-*p*NA in microplates according to the manufacturer’s protocol. The plates were read at OD 405 nm.

### Statistical analyses

Data were analyzed using SPSS (version 16.0; SPSS Inc., Chicago, IL). Statistical significance was evaluated using chi-square (χ^2^) and Mann-Whitney U-tests. Spearman rank correlation coefficients and Fisher’s exact tests were used to assess significant associations between different clinicopathological variables. Kaplan-Meier survival analysis, followed by the log-rank test for Pair-wise comparisons, was used to analyze the influence of STAT3 and EZH2 protein expression on overall survival of GC patients. A *P*-value of < 0.05 was considered significant and exact two-sided *P* values are reported.

## Additional file

Additional file 1: Table S1. Oligonucleotide primers for STAT3, EZH2, β-actin, reporter gene, EMSA, and siRNA of STAT3. **Table S2.** Correlation between STAT3 and EZH2 expression in GC. **Table S3.** STAT3 and EZH2 were associated with the clinical stage of the malignancy. **Table S4.** Multivariate analysis of factors associated with OS. **Figure S1.** The EZH2 promoter-Luc constructs designed for this study. (A) schematic map of the short and full-length EZH2 promoter-Luc constructs, the full length of construct contained three important STAT3 binding motifs (-1702/+52), the short construct (-1702/-447) didn’t contain this STAT3 binding region (-436/+52). (B) The promoter sequence of EZH2 was located from -436 to +52. The three STAT3-binding motifs are highlighted in blue. **Figure S2.** Deletion analysis of *EZH2* promoter activity. (A) Schematic maps of the *EZH2* promoter 5′-deletion constructs used as reporter plasmids. (B) Relative luciferase activity after transient transfection of the reporter plasmids into SGC7901 cells. The ratios between the luciferase activities induced by each reporter vector and pRL-TK were calculated. The data shown are means ± SE of triplicate experiments. **Figure S3.** Kaplan-Meier analysis showed the combination between p-STAT3 and EZH2 expression in overall survival of GC patients. **Figure S4.** Relative expression of STAT3 by knocking down with specific siRNA in SGC7901 by real-time PCR analysis (***p* < 0.01). **Figure S5.** Relative expression of STAT3 in GC cell lines by real-time PCR analysis (**p* < 0.05, ***p* < 0.01). **Figure S6.** Relative expression of EZH2 in GC cell lines by real-time PCR analysis (**p* < 0.05, ***p* < 0.01). **Figure S7.** Relative enrichment of EZH2 promoter fragment using real-time PCR analysis (**p* < 0.05). (DOCX 849 kb)

## Abbreviations

ChIP: Chromatin immunoprecipitation
DNMTs: DNA methyltransferases
DZNep: 3-deazaneplanocin A
EMSA: Electrophoretic mobility shift assay
EZH2: Enhancer of zeste homolog 2
FCM: Flow cytometry
GC: Gastric cancer
IHC: Immunohistochemistry
MTT: 3-(4,5-dimethylthiazol-2-yl) -2,5-diphenyltetrazolium bromide
RT-PCR: Reverse transcription-polymerase chain reaction
STAT3: Signal transducer and activator of transcription 3
