# Supplementary material for: STAT3 signaling drives EZH2 transcriptional activation and mediates poor prognosis in gastric cancer
Source: Mol Cancer. 2016 Dec 9;15:79. doi: 10.1186/s12943-016-0561-z (PMC5148878; doi:10.1186/s12943-016-0561-z)
Supplement: Additional file 1: Table S1. — Oligonucleotide primers for STAT3, EZH2, β-actin, reporter gene, EMSA, and siRNA of STAT3. Table S2. Correlation between STAT3 and EZH2 expression in GC. Table S3. STAT3 and EZH2 were associated with the clinical stage of the malignancy. Table S4. Multivariate analysis of factors associated with OS. Figure S1. The EZH2 promoter-Luc constructs designed for this study. (A) schematic map of the short and full-length EZH2 promoter-Luc constructs, the full length of construct contained three important STAT3 binding motifs (-1702/+52), the short construct (-1702/-447) didn’t contain this STAT3 binding region (-436/+52). (B) The promoter sequence of EZH2 was located from -436 to +52. The three STAT3-binding motifs are highlighted in blue. Figure S2. Deletion analysis of EZH2 promoter activity. (A) Schematic maps of the EZH2 promoter 5′-deletion constructs used as reporter plasmids. (B) Relative luciferase activity after transient transfection of the reporter plasmids into SGC7901 cells. The ratios between the luciferase activities induced by each reporter vector and pRL-TK were calculated. The data shown are means ± SE of triplicate experiments. Figure S3. Kaplan-Meier analysis showed the combination between p-STAT3 and EZH2 expression in overall survival of GC patients. Figure S4. Relative expression of STAT3 by knocking down with specific siRNA in SGC7901 by real-time PCR analysis (**p < 0.01). Figure S5. Relative expression of STAT3 in GC cell lines by real-time PCR analysis (*p < 0.05, **p < 0.01). Figure S6. Relative expression of EZH2 in GC cell lines by real-time PCR analysis (*p < 0.05, **p < 0.01). Figure S7. Relative enrichment of EZH2 promoter fragment using real-time PCR analysis (*p < 0.05). (DOCX 849 kb) [file 12943_2016_561_MOESM1_ESM.docx]

**Additional file 1**

**Table S1.** Oligonucleotide primers for STAT3, EZH2, β-actin, reporter gene, EMSA, and siRNA of STAT3.

**Table S2.** Correlation between STAT3 and EZH2 expression in GC.

**Table S3.** STAT3 and EZH2 were associated with the clinical stage of the malignancy.

**Table S4.** Multivariate analysis of factors associated with OS.

**Fig. S1.** The EZH2 promoter-Luc constructs designed for this study. (A) schematic map of the short and full-length EZH2 promoter-Luc constructs, the full length of construct contained three important STAT3 binding motifs (-1702/+52), the short construct (-1702/-447) didn’t contain this STAT3 binding region (-436/+52). (B) The promoter sequence of EZH2 was located from -436 to +52. The three STAT3-binding motifs are highlighted in blue.

**Fig.S2.** Deletion analysis of *EZH2* promoter activity. (A) Schematic maps of the *EZH2* promoter 5′-deletion constructs used as reporter plasmids. (B) Relative luciferase activity after transient transfection of the reporter plasmids into SGC7901 cells. The ratios between the luciferase activities induced by each reporter vector and pRL-TK were calculated. The data shown are means ± SE of triplicate experiments.

**Fig.S3.** Kaplan-Meier analysis showed the combination between p-STAT3 and EZH2 expression in overall survival of GC patients.

**Fig.S4.** Relative expression of STAT3 by knocking down with specific siRNA in SGC7901 by real-time PCR analysis (**p<0.01).

**Fig.S5.** Relative expression of STAT3 in GC cell lines by real-time PCR analysis (*p<0.05, **p<0.01).

**Fig.S6.** Relative expression of EZH2 in GC cell lines by real-time PCR analysis (*p<0.05, **p<0.01).

**Fig.S7.** Relative enrichment of EZH2 promoter fragment using real-time PCR analysis (*p<0.05).

**Table S1. Oligonucleotide primers**

| **Gene name** | **Sequence (5′-3′)** | **Size (bp)** |
| --- | --- | --- |
| STAT3 | F: GGAGGAGTTGCAGCAAAAAG | 322 |
|  | R: TGTGTTTGTGCCCAGAATGT |  |
| EZH2 | F: GTGGAGAGATTATTTCTCAAGATG | 289 |
|  | R: CCGACATACTTCAGGGCATCAGCC |  |
| β-actin | F: CCAAGGCCAACCGCGAGAAGATGAC | 587 |
|  | R: AGGGTACATGGTGGTGCCGCCAG |  |
| *EZH2*-promoter-Luc 1 (-1702/+52, Region 1) | F: CC *GCGCTC* CTCCGCCTTCTGGGTTCAAG  R: GCC *CTCGAG* CAGCCCAATCGCCATCGCTT | 1754 |
|  |  |  |
| *EZH2*-promoter-Luc 2 (-1702/-447, Region 2) | F: CC *GCGCTC* CTCCGCCTTCTGGGTTCAAG  R:GCC*CTCGAG* GGTGGTAACGGTTTTAACCG | 1256 |
|  |  |  |
| *EZH2*-promoter-Luc 3 (-436/+52, Region 3) | F:CC*GCGCTC* GAACTGGTTCAAACTTGGCTTC  R:GCC*CTCGAG* CAGCCCAATCGCCATCGCTT | 488 |
|  |  |  |
| EZH2-STAT3-1  (-373 ) | F: CC *GCGCTC* CGGTTCCCGCCAAGAGCCG  R:GCC*CTCGAG*CAGCCCAATCGCCATCGCTT | 425 |
|  |  |  |
| EZH2-STAT3-2  (-222) | F:CC*GCGCTC* GCCGTGTGTTCAGCGAAAGAA  R:GCC*CTCGAG* CAGCCCAATCGCCATCGCTT | 274 |
| EZH2-STAT3- 3  (-163) | F: CC *GCGCTC* GTCCCTTACAGCGAACCCCG  R:GCC*CTCGAG* CAGCCCAATCGCCATCGCTT | 215 |
|  |  |  |
| EZH2-STAT3-No-binding (-131) | F: CC*GCGCTC* CGCGCACGCTGCCAGTGC  R:GCC*CTCGAG* CAGCCCAATCGCCATCGCTT | 183 |
|  |  |  |
| *EZH2*-promoter-ChIP (-436/+48) | F: GAACTGGTTCAAACTTGGCTTC  R: CCAATCGCCATCGCTTTTAT | 484 |
| EMSA for STAT3-2 motif, wide type (-222/-197) | F:(Biotin/not)-GCCGTGTG*TT*CAGCG*AA*AGAACAAAG  R: CTTTGTTCTTTCGCTGAACACACGGC | 26 |
| EMSA for STAT3-2 motif mutant (-222/-197) | F: GCCGTGTG*GG*CAGCG*CC*AGAACAAAG  R: CTTTGTTCTGGCGCTGCCCACACGGC | 26 |
|  |  |  |
| STAT3 siRNA1 | F: GAGCUGCAAACAACUAUAC | 19 |
|  | R: GTATAGTTGTTTGCAGCTC |  |
| STAT3 siRNA2 | F: GCGACGUGAGGUAUAUGAC | 19 |
|  | R: GTCATATACCTCACGTCGC |  |
| STAT3 siRNA3 | F: GGAACAACAUUA GAACAGC | 19 |
|  | R: GCTGTTCTAATGTTGTTCC |  |
| Scrambled siRNA | F:GAATGCTGCGGAGGTAACT  R: AGTTACCTCCGCTGCATTC | 19 |

**Table S2. Correlation between STAT3 and EZH2 expression in GC**

| **EZH2 expression** | **STAT3 expression** | |  |
| --- | --- | --- | --- |
|  | **Low (-)** | **High (+)** | ***P*-value** |
| **Low (-)**  **High (+)** | 11  7 | 10  35 | 0.003 |
| Spearman correlation R = 0.373 | | | |

**Table S3. STAT3 and EZH2 were associated with the clinical stage of the malignancy**

| **TNM stage** | **EZH2 and STAT3 expression** | | | **Total** | **EZH2^+^/STAT3^+^(%)** | ***P*-value** |
| --- | --- | --- | --- | --- | --- | --- |
|  | **EZH2^-^/STAT3^-^** | **EZH2^-^/STAT3^+^ EZH2^+^/STAT3^-^** | **EZH2^+^/STAT3^+^** |  |  |  |
|  |  |  |  |  |  |  |
| I–II | 8 | 7 | 10 | 25 | 40% | 0.017 |
| III–IV | 3 | 10 | 25 | 38 | 65.70% |  |
| Total | 11 | 17 | 35 | 63 | 55.60% |  |

**Table S4. Multivariate analysis of factors associated with OS**

| **Features** | **HR** | **95.0% CIs** | | ***P*-value** |
| --- | --- | --- | --- | --- |
| TNM stage | 2.453 | 1.429 | 4.214 | 0.001 |
| EZH2 expression | 0.863 | 0.337 | 2.204 | 0.757 |
| STAT3 expression | 7.535 | 2.104 | 26.993 | 0.002 |
| Lymph node metastasis | 0.985 | 0.429 | 2.262 | 0.972 |
| Age | 0.989 | 0.959 | 1.020 | 0.481 |
| Gender | 0.678 | 0.311 | 1.476 | 0.327 |
| Distant metastasis | 0.977 | 0.604 | 1.582 | 0.926 |

**Figure S1**


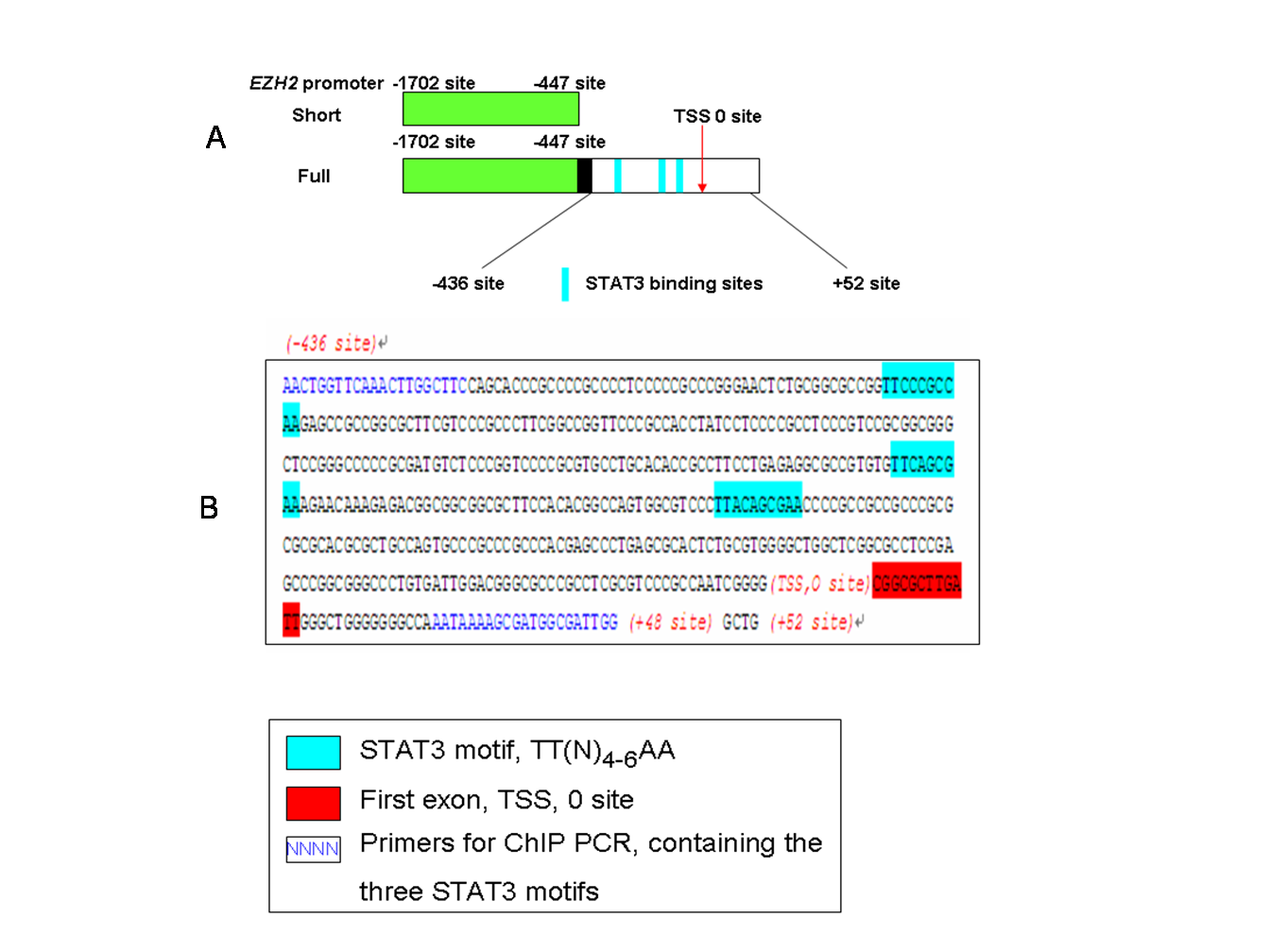


**Figure S2**


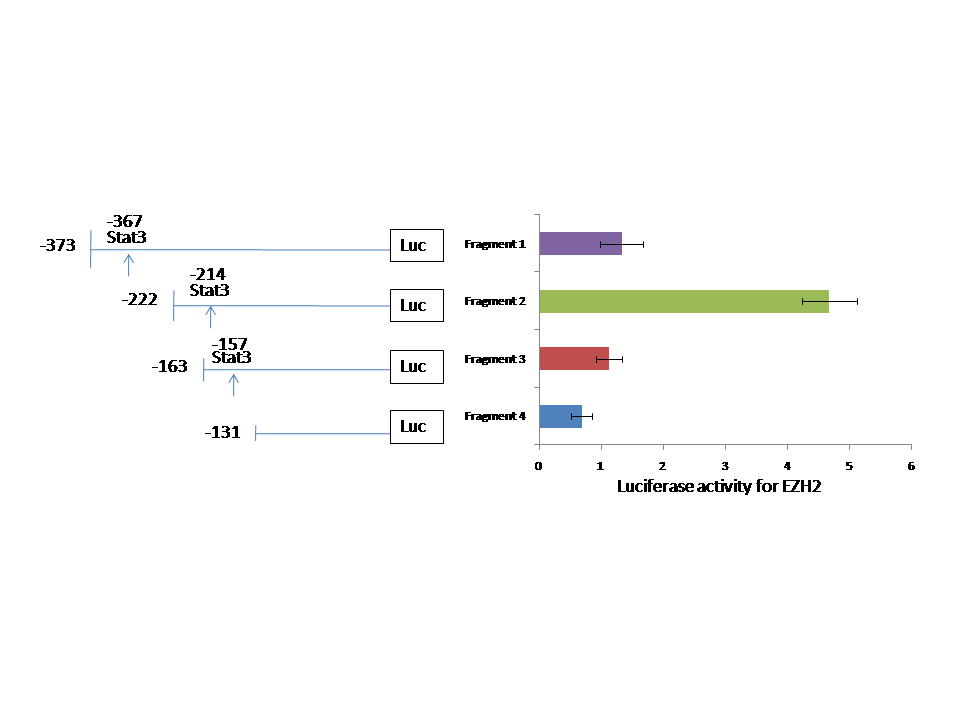


**Figure S3**


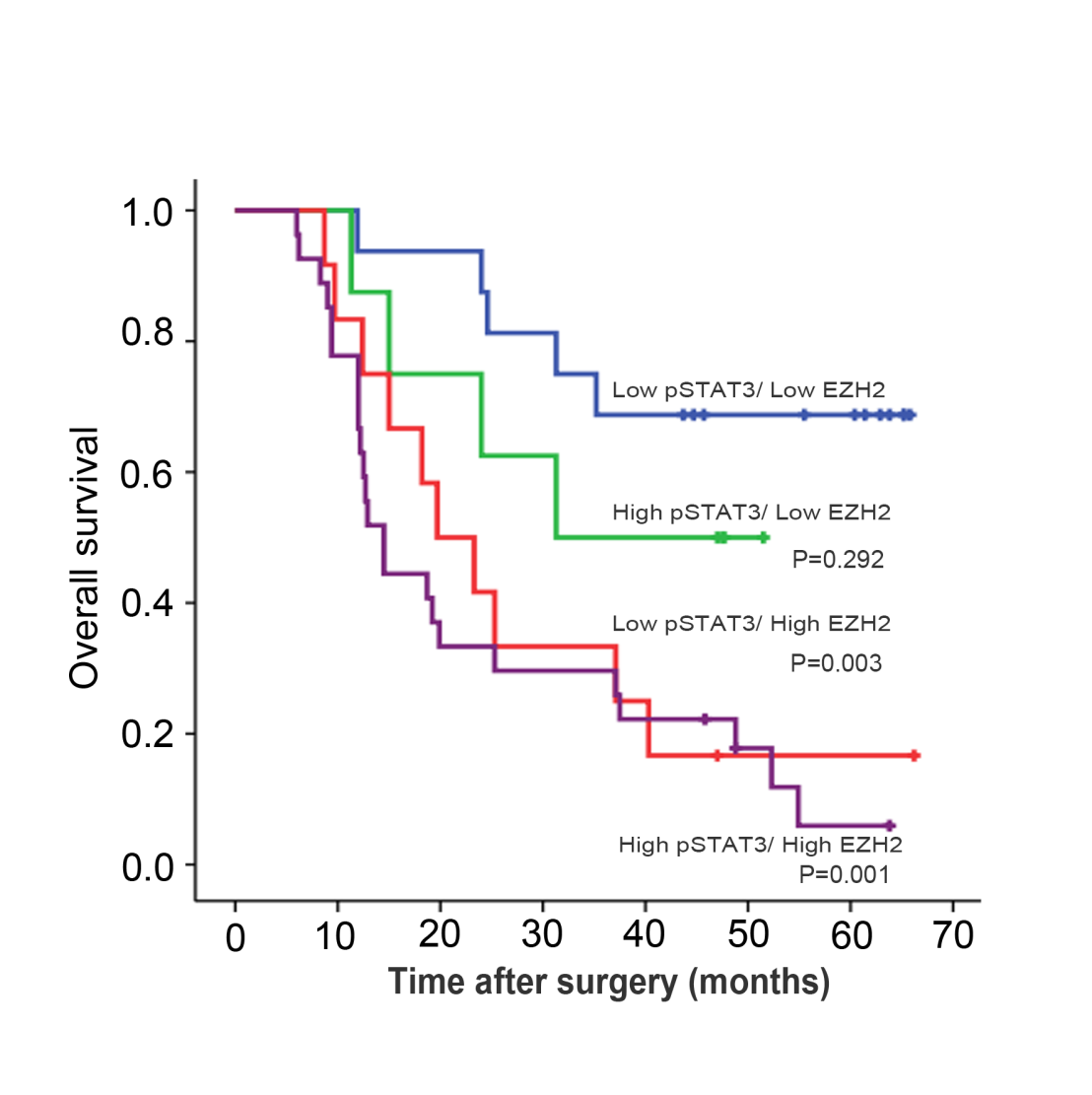


**Figure S4**





**Figure S5**

**

**

**Figure S6**

**

**

**Figure S7**

**

**
